# Unraveling the Spin-to-Charge Current Conversion Mechanism and Charge Transfer Dynamics at the Interface of Graphene/WS_2_ Heterostructures at Room Temperature

**DOI:** 10.1021/acsami.4c08539

**Published:** 2024-10-02

**Authors:** Rafael O. Cunha, Yunier Garcia-Basabe, Dunieskys G. Larrude, Matheus Gamino, Erika N. Lima, Felipe Crasto de Lima, Adalberto Fazzio, Sergio M. Rezende, Antonio Azevedo, Joaquim B. S. Mendes

**Affiliations:** † Departamento de Física, Universidade Federal de Viçosa, Viçosa 36570-900, Minas Gerais, Brazil; ‡ Centro Interdisciplinar de Ciências da Natureza, Universidade Federal da Integração Latino-Americana, Foz do Iguaçu 85867-970, Paraná, Brazil; § Escola de Engenharia, Universidade Presbiteriana Mackenzie, São Paulo 01302-907, Brazil; ∥ Departamento de Física, Universidade Federal do Rio Grande do Norte, Natal 59078-900, Rio Grande do Norte, Brazil; ⊥ Instituto de Física, Universidade Federal de Mato Grosso, 78060-900 Cuiabá, Mato Grosso, Brazil; # Ilum School of Science, Brazilian Center for Research in Energy and Materials (CNPEM), 13083-970 Campinas, São Paulo, Brazil; ¶ Departamento de Física, Universidade Federal de Pernambuco, 50670-901 Recife, Pernambuco, Brazil

**Keywords:** spintronics, transition-metal dichalcogenides, graphene, spin-to-charge current conversion, charge transfer dynamics, Rashba spin−orbit coupling

## Abstract

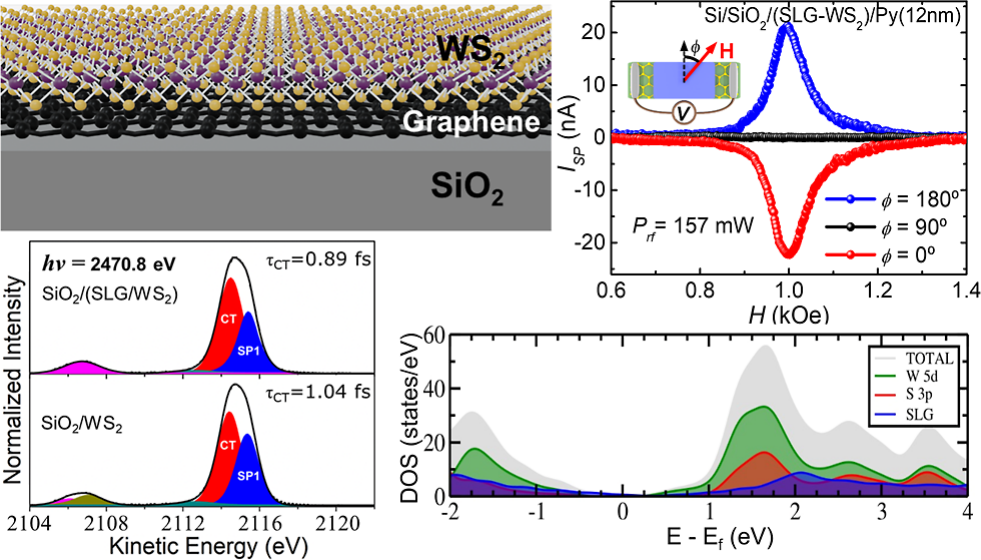

We report experimental investigations of spin-to-charge current conversion and charge transfer (CT) dynamics at the interface of the graphene/WS_2_ van der Waals heterostructure. Pure spin current was produced by the spin precession in the microwave-driven ferromagnetic resonance of a permalloy film (Py=Ni_81_Fe_19_) and injected into the graphene/WS_2_ heterostructure through a spin pumping process. The observed spin-to-charge current conversion in the heterostructure is attributed to the inverse Rashba–Edelstein effect (IREE) at the graphene/WS_2_ interface. Interfacial CT dynamics in this heterostructure was investigated based on the framework of the core-hole clock (CHC) approach. The results obtained from spin pumping and CHC studies show that the spin-to-charge current conversion and charge transfer processes are more efficient in the graphene/WS_2_ heterostructure compared to isolated WS_2_ and graphene films. The results show that the presence of WS_2_ flakes improves the current conversion efficiency. These experimental results are corroborated by density functional theory (DFT) calculations, which reveal (i) Rashba spin–orbit splitting of graphene orbitals and (ii) electronic coupling between graphene and WS_2_ orbitals. This study provides valuable insights for optimizing the design and performance of spintronic devices.

## 1 Introduction

Scientific research consistently seeks to discover new phenomena and re-evaluate existing ones through the lens of advancing technology. In the field of materials physics, spintronics has attracted significant attention from several research groups, paving the way for new discoveries and phenomena, such as the spin Hall effect, spin current manipulation, and orbitronics.^1−5^ The investigation of nanoscale material structures is fundamental to these efforts, particularly in preserving spin memory. Although thin films have traditionally dominated this area, a class of two-dimensional materials, including transition-metal dichalcogenides (TMDs) and graphene, has emerged as a central point of interest. These materials are very promising, especially in the domain of device fabrication for applications spanning sensors, electronics, spintronics, and optoelectronics.^6^

In spintronics, graphene has emerged as an attractive candidate for spin current transport despite its inherent low spin–orbit coupling (SOC), resulting in limited conversion of spin current to charge current.^7−9^ However, it has been explored in conjunction with ferromagnetic materials to induce SOC by a proximity effect within the graphene layer.^10^ On the other hand, TMDs exhibit strong SOC but moderate spin mobility.^11^ In this work, we take advantage of these two characteristics by exploiting a heterostructure composed of graphene and TMD tungsten disulfide (WS_2_). This combination induces strong spin–orbit coupling in graphene through the proximity effect, facilitating efficient spin current conduction and spin current-to-charge current conversion.^9,12^ Exploring the physics of these materials has been greatly facilitated by advances in nanofabrication processes. However, manipulating two-dimensional structures, such as through nanolithography, requires sophisticated techniques.^13^ In our research, we employ a simplified fabrication approach for the heterostructure, optimizing time and costs to increase efficiency in large-scale production.

It is known that the proximity effect induced in graphene increases the efficiency of converting spin current into charge current through the inverse Rashba–Edelstein effect (IREE).^12,14^ This work aims to elucidate the underlying mechanisms that drive this efficiency increase observed in spin-pumping measurements by microwave-driven ferromagnetic resonance (FMR) at room temperature. Specifically, we investigate the electronic coupling between the S 3p states of WS_2_ and the electronic states of the conduction band of graphene. Our investigation of the charge transfer (CT) dynamics in this heterostructure is complemented by ab initio calculations employing density functional theory (DFT). The results obtained in this paper provide valuable insights into optimizing the design and performance of spintronic devices based on 2*D*/2D van der Waals (vdW) heterostructures.

## 2 Materials and Methods

**Figure 1:**
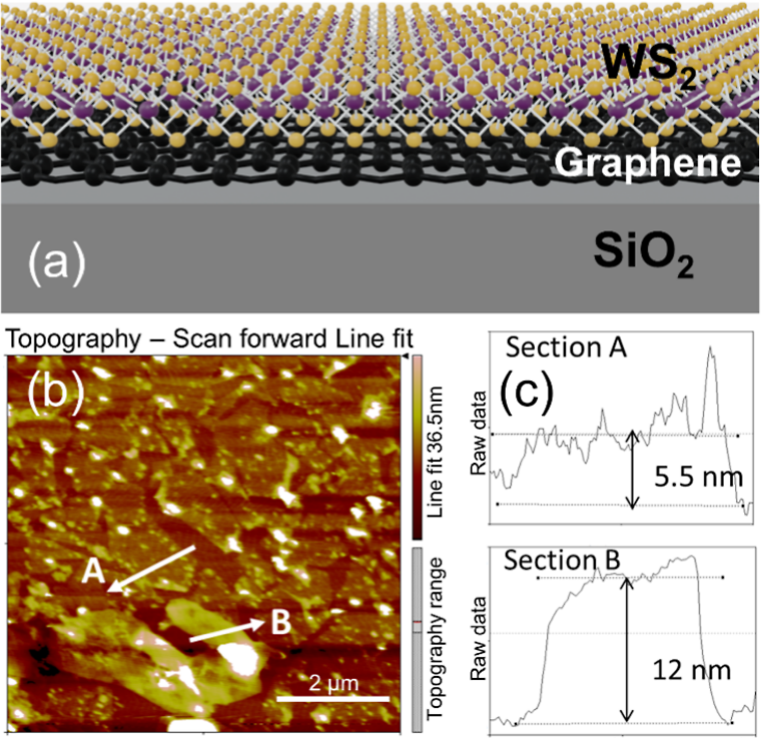
(a) Illustrative schematic diagram of the SLG/WS_2_ heterostructure on a SiO_2_ substrate (gray). Carbon atoms of the SLG are depicted as black dots, while sulfur and tungsten atoms of WS_2_ are represented by yellow and purple dots, respectively. (b) Atomic force microscopy height image of the SLG/WS_2_ heterostructure. (c) Height profile in regions A and B of (b).

The heterostructure comprising a single layer of graphene (SLG) and a layer of tungsten disulfide (WS_2_), henceforth referred to as SLG/WS_2_, was synthesized by first transferring CVD graphene onto a Si/SiO_2_ substrate using a well-established procedure known in the literature,^10,15^ and then WS_2_ was subsequently deposited onto the transferred SLG sheet. This deposition was obtained through the spin coating method at 700 rpm for 60 s using a solution of WS_2_ nanoflakes dispersed in ethanol/water (26 mg/L).^16^Figure 1a shows an illustrative scheme of the SLG/WS_2_ heterostructure placed on a SiO_2_ substrate.

The morphology and thickness of the SLG/WS_2_ heterostructure were investigated by atomic force microscopy (AFM). Figure 1b reveals significant spatial heterogeneity in the contact region between the WS_2_ nanosheets and the graphene layer, with a film surface roughness of 4.3 nm. The height profile shows thickness variation ranging from 5.5 to 12 nm, indicating the presence of 5–15 layers of WS_2_ over SLG.

## 3 Results and Discussion

### 3.1 Sample Characterization

**Figure 2:**
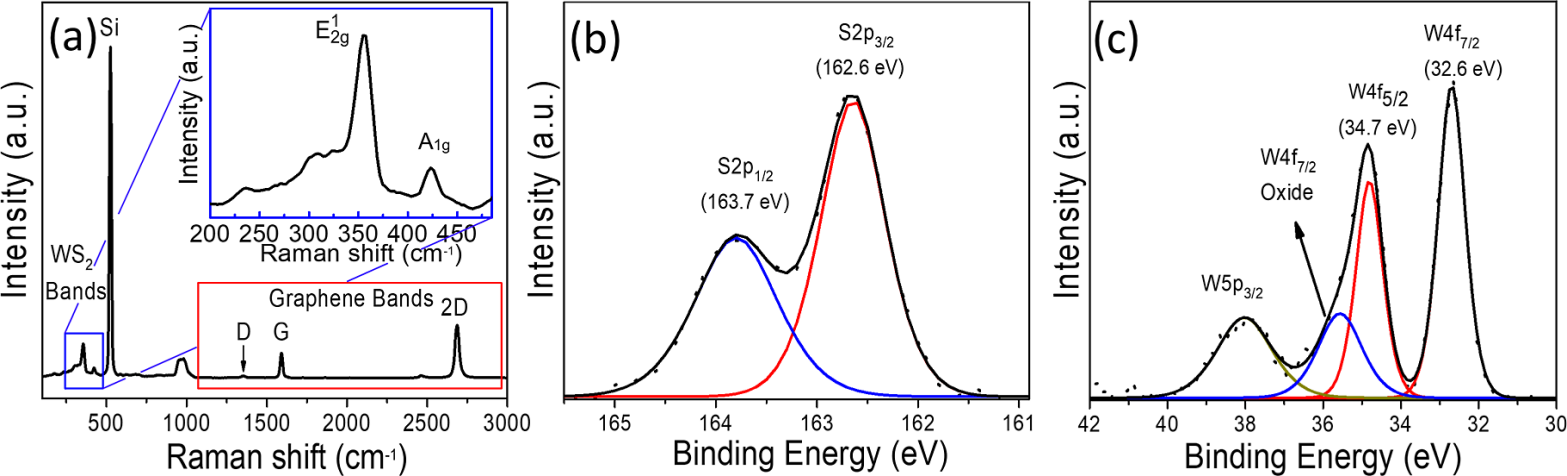
(a) Raman spectrum of the SLG/WS_2_ heterostructure. The inset shows E_2g_^1^ and A_1g_ Raman modes of WS_2_ in detail. High-resolution XPS spectra of the SLG/WS_2_ heterostructure: (b) S 2p and (c) W 4f core levels. Blue and red lines represent the fittings of XPS spectra.

To investigate the crystalline phase of the heterostructure, Raman spectra were collected at room temperature. Figure 2a shows the Raman spectrum of the SLG/WS_2_ heterostructure, revealing characteristic vibrational modes typical of graphitic materials, namely, the D, G, and 2D peaks.^17^ The G-band corresponds to the in-plane optical mode arising from the bond stretching of graphitic sp^2^ carbon atoms. The D-band originates from the lattice defect and distortion, while the 2D-band is attributed to inter- and intragale scattering processes. The ratio of intensities I(2D)/I(G) is ∼1.5, and the FWHM of the 2D band obtained from the Lorentzian fit is 29.5 cm^–1^, which is characteristic of the graphene monolayer.^18^ The two peaks observed at 357 and 423 cm^–1^ (see the inset in Figure 2a) correspond to the in-plane E_2g_^1^ and out-plane A_1g_ vibrational modes of 2H-WS_2_, respectively.^19−21^ These values indicate that WS_2_ has a semiconductor phase.

In addition to structural characterizations, Figure 2b,c shows the S 2p and W 4f high-resolution X-ray photoelectron spectroscopy (XPS) spectra of SLG/WS_2_, respectively. S 2p XPS spectra (see Figure 2b) are characterized by the S 2p_3/2_ (162.6 eV) and 2p_1/2_ (163.7 eV) spin–orbital doublet. On the other hand, the W 4f XPS spectrum (see Figure 2c) is characterized by two peaks at 32.6 and 34.7 eV, corresponding to the W 4f_7/2_ and W 4f_5/2_ components, respectively, indicative of the 2H-WS_2_ phase.^22,23^ Additionally, a peak at 35.8 eV corresponding to W 4f_7/2_ of W oxidized species is observed in this spectrum.^23^

### 3.2 Spin-Pumping Experiments

**Figure 3:**
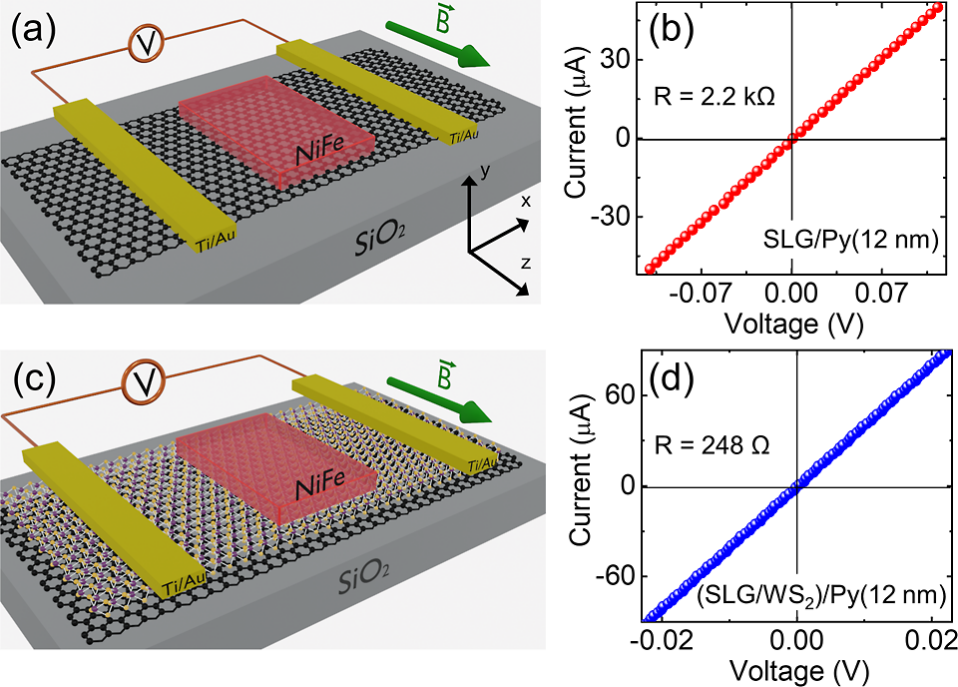
Illustration of the (a) SLG/Py (12 nm) and (c) (SLG/WS_2_)/Py (12 nm) structures along with Ti (30 nm)/Au (5 nm) electrodes to ensure ohmic contact. In both samples, the Py film was deposited in the center of the sample without physical contact with the electrodes. The *I*–*V* curve demonstrates that the electrical contact between the electrodes and the (b) SLG film and (d) (SLG/WS_2_) heterostructure has ohmic behavior.

For the spin-pumping experiments, a 12 nm thick Py (Permalloy, Ni_81_Fe_19_) film was sputter-deposited in an ultrahigh vacuum chamber with a base pressure of 4 × 10^–8^ mbar onto both a SLG film and the SLG/WS_2_ heterostructure, forming islands at the center of each sample, as shown in the schematics of Figure 3a,c, respectively. To ensure ohmic contact, Ti (30 nm)/Au (5 nm) strips were deposited by sputtering on the ends of the samples. Two thin copper electrodes were fixed to the Ti/Au strips by means of silver paint and connected to the nanovoltmeter, as shown in detail in Figure 3a,c. It is important to note that the electrical contacts are not in contact with the Py film to prevent current shunting. Figure 3b,d shows the *I–V* curves for the samples SLG/Py and (SLG/WS_2_)/Py, respectively, demonstrating that the electrical contact between Ti/Au and the electrodes is ohmic. The resistance of the SLG film is R = 2.2 kΩ, significantly higher than the electrical resistance of the SLG/WS_2_ heterostructure, with R = 248 Ω. The significant reduction of the electrical resistance of SLG/WS_2_ may be attributed to the achieved high electron-doping density, thus a significant reduction of the Schottky barrier.^24−27^

**Figure 4:**
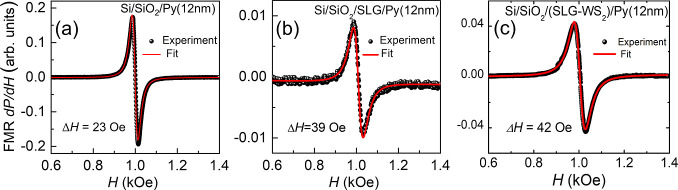
Panels show the field scan FMR microwave absorption derivative spectra, , at a frequency of 9.4 GHz for three samples: (a) pure 12 nm thick Py film, (b) SLG/Py, and (c) (SLG/WS_2_)/Py, with the magnetic field applied in the film plane.

Figure 4 shows the FMR absorption derivative of the Py layer deposited directly on the Si substrate as well as on the SLG film and the SLG/WS_2_ heterostructure. These measurements were performed with an incident microwave power of 157 mW, at 9.4 GHz and a resonance field of *H*_R_ = 1 kOe. The FMR absorption line has the shape of a Lorentzian derivative with half-width at half-maximum (HWHM) line width of Δ*H*_Py_ = 23 Oe for the Py film on the Si substrate, as shown in Figure 4a. Figure 4b shows the absorption line for a sample composed of SLG/Py without the WS_2_ sheet. The HWHM line width for this sample is Δ*H*_SLG/Py_ = 39 Oe. The heterostructure compound by (SLG/WS_2_)/Py has the HWHM line width of  Oe, as shown in Figure 4c. These results show that due to the spin pumping process, additional damping is produced with the presence of single-layer graphene and the SLG/WS_2_ heterostructure^28,29^ of δ*H*_SLG_ ≡ (Δ*H*_SLG/Py_ – Δ*H*_Py_) = 16 Oe and  Oe, respectively. Similar effects have been observed in bilayers formed by different materials in atomic contact with Py, such as normal metals/Py,^30−34^ semiconductors/Py,^35−40^ graphene/Py,^10,41,42^ and topological insulators/Py.^43−45^

A spin current density  is generated by the precessing magnetization *M⃗* in the Py layer (FM layer), which flows perpendicularly to the interfaces with nonmagnetic materials (NMs), such as the SLG film or the SLG/WS_2_ heterostructure. This spin current exhibits transverse spin polarization σ̂, defined by . Here, *g*_eff_^↑↓^ is the real part of the effective spin mixing conductance at the interface that takes into account both the spin-pumped and back-flow spin currents,^46^ while *M*_s_ denotes the saturation magnetization. Expressing the magnetization as , where *m*_*x*_,*m*_*y*_ ≪ *M*_*z*_, the spin current density that is pumped through the FM/NM interface can be written in units of angular moment per area per time as
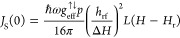
1Here, ω represents the microwave excitation frequency, *h*_rf_ is the amplitude of the microwave magnetic field,  is the Lorentzian function,  indicates the ellipticity factor of the precession cone, and Δ*H* is the FMR line width at HWHM. The pure spin current flowing through the FM/NM interface consists of electrons with opposite spins moving in opposite directions. As it diffuses into the NM, a spin accumulation is created given by . Here, *t*_NM_ and λ_S_, respectively, denote the thickness and spin diffusion length of the NM layer, such as SLG or SLG/WS_2_.

In bulk states, the inverse spin Hall effect (ISHE) converts a portion of the spin current density into a transverse charge current density given by , where θ_SH_ represents the spin Hall angle, quantifying the efficiency of the spin-to-charge current conversion process, and σ̂ represents the spin polarization direction. Integrating the charge current density yields the spin pumping current, , where *w* is the width of the NM (the distance between electrodes). Similarly, surface states can exhibit analogous phenomena via the inverse Rashba–Edelstein effect (IREE). Through measurements of the FMR absorption and spin-pumping voltage, one can extract material parameters by investigating these two distinct phenomena (ISHE and IREE). However, our focus in this work is to explore the influence of WS_2_ in two-dimensional structures on the enhancement of spin-charge conversion. Thus, we concentrate on the IREE phenomenon.

**Figure 5:**
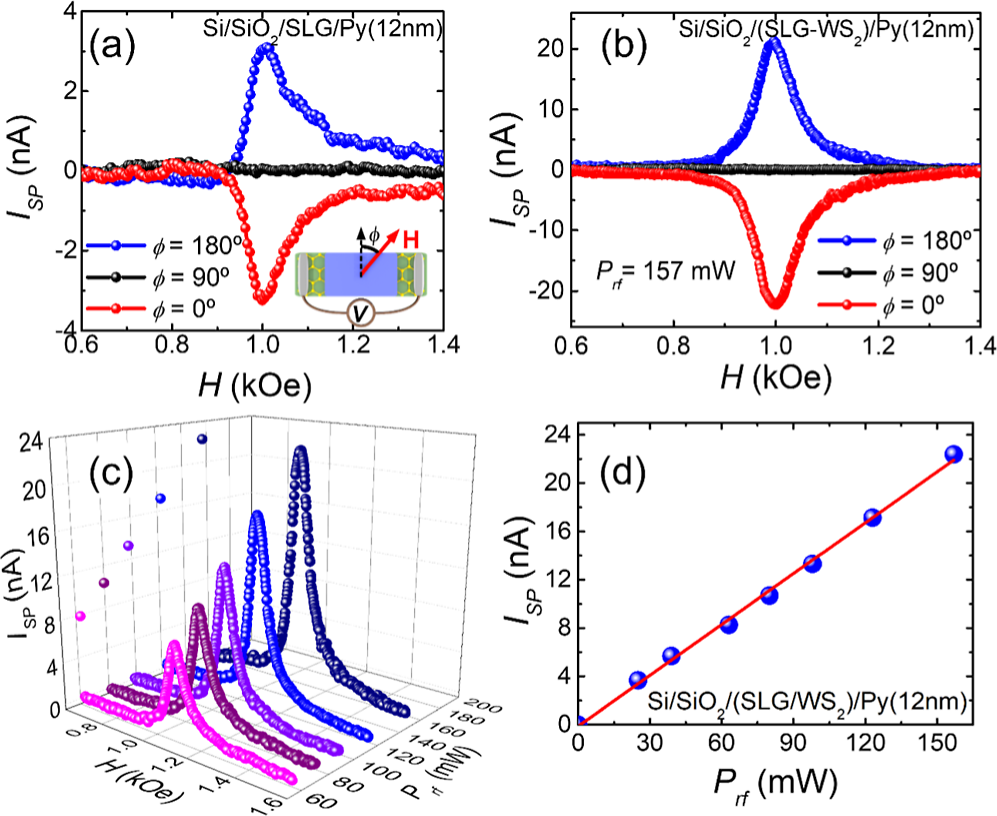
Field scan of the spin pumping current *I*_SP_ measured in (a) SLG/Py and (b) (SLG/WS_2_)/Py for angles of ϕ = 0, 90, and 180° with a magnetic field applied in the plane, as shown in the inset, under 157 mW incident microwave power at a microwave frequency of 9.4 GHz. (c) Field scans of |*I*_SP_| for different values of the incident microwave power for ϕ = 180° and (d) peak current value as a function of the rf excitation power measured on the (SLG/WS_2_)/Py sample.

To make a comparison between the spin pumping responses of the SLG/WS_2_ heterostructure and SLG, we use the spin pumping current , which is a more significant physical parameter since *V*_SP_ is proportional to the electric resistance *R* and depends on the electrical contact characteristics. Figure 5a shows the current *I*_SP_ plotted as a function of the applied magnetic field *H*, with a microwave power of 157 mW. As the field sweeps, a dc voltage *V*_SP_ is directly measured at the FMR field value. To achieve this, we employed a nanovoltmeter connected to the Ti/Au electrodes via copper wires. In the case of the SLG/Py sample, resistance *R* is measured to be 2.2 kΩ. The SP current is positive for ϕ = 180° (blue curve), changes sign with field inversion (ϕ = 0°, red curve), and vanishes for the field along the sample strip ϕ = 90° (black curve). The contributions of galvanomagnetic or spin rectification effects,^31,47^ generated by the Py layer itself, can be neglected. This is due to the Py film being deposited on the SLG (as well as in the SLG/WS_2_ heterostructure) in a manner that isolates it as an island between the electrical contacts, thus preventing any shunting of the charge current. Figure 5b shows the field dependence of *I*_SP_ for the (SLG/WS_2_)/Py sample, where *R* = 248 Ω. Here, one can observe behavior similar to that of the sample without WS_2_. Comparing the maximum values of the currents *I*_SP_ measured at the resonance in Figure 5a,b, both measured at the same rf power *P*_rf_ = 157 mW, we can observe that *I*_SP_^peak^ of the (SLG/WS_2_)/Py sample is approximately 7 times larger than *I*_SP_^peak^ of the SLG/Py sample without the WS_2_ sheets. This result confirms that the presence of WS_2_ significantly enhances spin-to-charge interconversion, indicating that the SLG/WS_2_ heterostructure is substantially more efficient in converting the spin current into the charge current compared to a single layer of graphene. This large efficiency can be attributed to the large SOC present in transition metal dichalcogenides such as WS_2_. A theoretical study on this phenomenon will be discussed later in this work.

Figure 5c shows the behavior of the spin pumping current for different rf powers (*P*_rf_) as a function of the applied magnetic field at ϕ = 0°. The maximum value of the spin pumping current (*I*_SP_^peak^) presents a linear dependence with the rf power (see Figure 5d), such that nonlinear effects are not present in the excitation regime used in the experiments.

As previously mentioned, to investigate the enhancement efficiency of the spin-charge conversion process in SLG/WS_2_ heterostructures, we will focus on the IREE phenomena, particularly suitable for the two-dimensional characteristics of our samples. We consider that due to the spin pumping process in Py, a 3D spin current density *J*_S_ is generated, and it is converted by IREE into a lateral charge current with 2D current density *j*_C_ when it flows into the SLG film or the SLG/WS_2_ heterostructure. The relationship between density *j*_C_ and *J*_S_ is given by *j*_C_=(2*e*/*ℏ*)λ_IREE_*J*_S_, where the IREE coefficient λ_IREE_ quantifies the conversion process. Here, the charge current density *j*_C_ is given by *j*_C_ = *I*_SP_^peak^/*w*, where *w* = 3.0 mm is the size of the samples (the distance between electrodes). At the interface, the spin current density produced by the FMR spin pumping *J*_S_ is calculated by using eq 1. Combining the current density equations *j*_C_ and *J*_S_, an expression for the IREE coefficient can be derived in terms of the measured charge current peak value
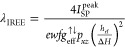
2The relationship between the amplitude of the microwave field *h*_rf_, in Oe, and the incident power *P*_i_, in watts, is given by , calculated for a microwave cavity operating in the transverse electric (TE102) mode, made with a shorted standard X-band rectangular waveguide at a frequency of 9.4 GHz, with a *Q* factor of 2000.^45^ For *P*_i_ = 157 mW, this gives *h*_rf_ = 0.704 Oe. Δ*H* is the line width at HWHM of the sample. The effective spin mixing conductance is given by ,^10,34,45^ which depends on the saturation magnetization *M*_S_ and thickness *t*_Py_ of Py, the increase in the line width at HWHM δH in Py by the additional damping coming from the spin pumping process due to the contact with the SLG film and SLG/WS_2_ heterostructure, and the microwave frequency ω/2π = 9.4 GHz. The microwave frequency is related to the resonance field by the Kittel equation , where γ is the gyromagnetic ratio and 4π*M*_eff_ is the effective magnetization. The effect of the perpendicular anisotropy in thin films causes the effective magnetization to be smaller than saturation magnetization. The magnetic anisotropy of Py is small. We obtain 4π*M*_eff_ = 9.22 kG, consistent with values for 12 nm thick Py at room temperature,^31,48^ using γ = 2.94 GHz/kOe, corresponding to a *g*-factor for Py of 2.1 and the measured *H*_R_ = 1.0 kOe (see Figure 4). The thickness of Py in all samples is *t*_Py_ = 12 nm. The additional damping in SLG/Py and (SLG/WS_2_)/Py samples are, respectively, δ*H*_SLG_ = 16 Oe and  Oe, which result in correspondent *g*_eff_^↑↓^ values of 2.45 × 10^18^ and 2.91 × 10^18^ m^–2^.

Finally, using *I*_SP_^SLG/Py^ = (3.11 ± 0.04) nA, *g*_eff_^↑↓^ = 2.45 × 10^18^ m^–2^, and Δ*H*_SLG/Py_ = 39 Oe, we obtain *j*_C_^SLG/Py^ = 1.04 × 10^–6^ A/m, *J*_S_^SLG/Py^ = 9.29 × 10^4^ A/m^2^, and, consequently, λ_IREE_^SLG/Py^=(0.011 ± 0.002) nm for the SLG/Py sample. Similarly, to the (SLG/WS_2_)/Py sample, using *I*_SP_^(SLG/WS2)/Py^=(22,26 ± 0.07) nA, *g*_eff_^↑↓^ = 2.91 × 10^18^ m^–2^, and Δ*H*_(SLG/WS2)/Py_ = 42 Oe, we obtain *j*_C_^(SLG/WS2)/Py^ = 7.42 × 10^–6^ A/m, *J*_S_^(SLG/WS2)/Py^ = 9.52 × 10^4^ A/m^2^, and λ_IREE_^(SLG/WS2)/Py^= (0.078 ± 0.005) nm. The deviations in the values of λ_IREE_ were by taking into account the variation of *I*_SP_ measured at ϕ = 0° and 180°.

The λ_IREE_ value extracted from the sample with WS_2_ sheets is seven times larger than that observed in the sample without the presence of the TMD and comparable or even larger than the values reported for several topological insulators.^13,45,49−53^ It is important to emphasize that the magnitude of the IREE coefficient also depends on the sample preparation processes in addition to the intrinsic properties of graphene and WS_2_. In previous studies of spin pumping works based on two-dimensional materials, the increase in the efficiency of the spin conversion is associated with the interface property.^10,13,45^ Thus, the spin conversion efficiency can be compromised due to structural defects in the fabrication process of heterostructures. Therefore, a detailed investigation of understanding the correlation of the spin-charge conversion efficiency and surface roughness and interfacial conditions would be desirable. The effective spin mixing conductance *g*_eff_^↑↓^ shows negligible variation, given that the δH line width increment is practically identical in both samples. Therefore, the key factor influencing λ_IREE_ lies in the magnitude of charge current *j*_C_ resulting from the conversion of *J*_S_. Clearly, the presence of WS_2_, particularly when combined with SLG, significantly increases the spin-to-charge current conversion efficiency of the investigated heterostructure.

A detailed investigation, as described below, investigated the electronic coupling between the S 3p states of WS_2_ and the electronic states of the conduction band of SLG. This investigation aimed to understand the underlying mechanisms that drive the observed increase in the conversion efficiency of the spin current to the charge current, as revealed in the spin-pumping measurements.

### 3.3 Charge Transfer Dynamics Analysis

The electronic coupling between WS_2_ and graphene was investigated by considering the charge transfer (CT) dynamics in SLG/WS_2_ heterostructures. This transfer was explored through a combination of techniques, including X-ray absorption near edge structure (S K-edge NEXAFS) and resonance Auger spectroscopy (S–K L_2,3_L_2,3_ RAS), employing the core-hole clock (CHC) approach. Specifically, the interfacial CT dynamics in the SLG/WS_2_ heterostructure are examined for excited electrons from the S 1s to S 3p_x,y_ and S 3p_z_ electronic states within the framework of the CHC approach using S–K L_2,3_L_2,3_ RAS spectroscopy.

The CHC method is element-sensitive and orbital-dependent, enabling precise investigation of the CT dynamics on the femtosecond and subfemtosecond time scale. This technique takes advantage of the generation of a core-hole interaction, occurring when a core electron is photoexcited to unoccupied states by X-ray photon absorption. Two possible types of decay channels can be observed depending on (i) whether the excited electron remains atomically localized during the core-hole lifetime or (ii) if it is delocalized to the surrounding environment of the atom before core-hole refilling. In the first case, two distinct final states can be reached: the spectator two-hole and one electron final states (2h1e), where the excited electron remains on the site and an electron in another level fills the core hole, and the participator one-hole final state (1h), wherein the excited electron can take part in the decay process, either through Auger electron emission or core-hole filling. In the second case, the excited electron is delocalized out of the atom during the core-hole lifetime, reaching two holes (2h) in the valence band, final state. This process is energetically similar to normal Auger decay due to a direct core-level photoionization process and is called the charge transfer (CT-Auger) channel, while the spectator and participator are usually called the “Raman” decay channel. The charge transfer time (τ_CT_) is estimated from the branching ratio between Raman and CT-Auger decay channels and using the lifetime of the created core hole as an internal clock. In the CHC approach, the accessible CT time scale range is 0.1τ_CH_ < τ_CT_ < 10τ_CH_.^54^ A schematic representation of the CHC approach described above is shown in the Supporting Information Figure S1.

**Figure 6:**
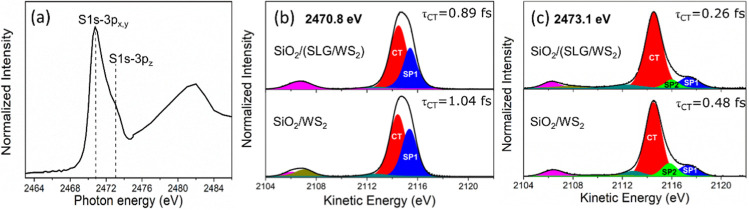
(a) S K-edge NEXAFS spectra of the SLG/WS_2_ heterostructure collected at a 45° incidence angle. The main S 1s–3p electronic transitions within the resonance are represented by the dashed lines. S–K L_2,3_L_2,3_ RAS spectra deconvoluted in Raman spectators SP1 (blue feature) and SP2 (green) and CT (red curve) decay channels collect at photon energies (b) 2470.8 eV corresponding to S 1s–3p_*x*,*y*_ transition and (c) 2473.1 eV corresponding to S 1s–3p_*z*_ transition.

The S K-edge NEXAFS spectrum of the SLG/WS_2_ heterostructure is shown in Figure 6a. Inside the S K-edge NEXAFS resonance region (below the sulfur ionization threshold of 2474.7 eV), this spectrum is characterized by two main features arising from electron transitions originating from the S 1s core level to 3p unoccupied states. The primary feature, appearing at the maximum of the resonance photon energy (2470.8 eV), is attributed to transitions from S 1s to 3p_*x*,*y*_ (in-plane) electronic states, while the second feature, localized at 2473.1 eV, is associated with electronic transitions from S 1s to 3p_z_ (out-plane) states.^22,55,56^

Figure 6b,c shows the S–K L_2,3_L_2,3_ RAS spectra of the SLG/WS_2_ heterostructure obtained at 2470.8 and 2473.1 eV photon energies, which correspond to S 1s–3p_*x*,*y*_ and S 1s–3p_z_ transitions, respectively. For comparison, the S–K L_2,3_L_2,3_ RAS for a WS_2_ sample deposited directly on a SiO_2_ substrate without the single layer of graphene, collected at these same photon energies, was also included in this figure. S–K L_2,3_L_2,3_ RAS spectra are formed of ^1^S and ^1^D Auger multiplets of the S 3p states.^57^ The distinction between Raman (spectator SP) and CT-Auger decay channels in the RAS spectra is based on the fact that due to energy conservation, the kinetic energy (KE) of the Raman decay channels increases when photon energy is tuned across the resonance, while the kinetic energy of the CT-Auger contribution is independent of the excitation energy. The dependency of the electron kinetic energy with excitation energy for these decay channels is presented in Figure S2.

In order to apply the CHC approach for the SLG/WS_2_ heterostructure, the RAS spectra were deconvoluted in Raman spectator components (SP1 and SP2) and CT-Auger contributions, as shown in Figure 6b,c. The fitting analysis of RAS spectra was performed following the method reported by Garcia-Basabe et al.^57^ Pseudo-Voight profile functions (a linear combination of Lorentzian and Gaussian functions) implemented in the CasaXPS software package (version 2.3.2) were used. The background was corrected by using a Shirley function. The C 1s (C–C) photoemission line localized at a binding energy of 284.5 eV was used to monitor the possible surface charge effects (shifts in electron energy). The standard deviation obtained from this RAS curve fitting procedure was ∼ 5%.

The spectra collected at 2470.8 eV are characterized by the CT-Auger contribution appearing at a constant kinetic energy of 2114.5 eV (red curve) and the spectator contribution SP1 (blue curve) representing the electron localized in 3p_*x*,*y*_ states. Furthermore, an additional spectator contribution, SP2 (green curve), was observed in the RAS spectra collecting 2473.1 eV, corresponding to electrons localized in 3p_z_ states. The main fitting parameters of the RAS spectra to SiO_2_/WS_2_, such as kinetic energy (KE), full width at half-maximum (FWHM), and area (A), are presented in Table S1 in the Supporting Information.

The τ_CT_ values for the SLG/WS_2_ heterostructure and WS_2_ sample on SiO_2_ were calculated using τ_CT_ = τ_CH_*x*(*I*_Raman_/*I*_CT-Auger_), where I_Raman_ and I_CT-Auger_ are the integral intensities of spectators (SP1 and SP2) and CT-Auger decay channels, respectively. The S 1s core hole has a characteristic lifetime (τ_CH_) of 1.27 fs.^58^ Thus, the τ_CT_ for the SLG/WS_2_ heterostructure was calculated for S 1s–3p_*x*,*y*_ (2470.8 eV) and S 1s–3p_*z*_ (2473.1 eV) transitions of 0.89 ± 0.10 and 0.26 ± 0.10 fs, respectively. On the other hand, the τ_CT_ values of 1.04 ± 0.21 and 0.48 ± 0.20 fs were obtained for the WS_2_ sample on SiO_2_. Here, we found that the electron delocalization from the S 3p electronic states is significantly faster in the SLG/WS_2_ heterostructure compared to the WS_2_ without the SLG system, mainly to electrons excited to 3p_*z*_ (out-plane) states, which is two times faster in the SLG/WS_2_ heterostructure. This result is evidence of electronic coupling between the S 3p electronic states of WS_2_ and the electronic states of the graphene conduction band, favoring interfacial electron transfer.

These results of charge transfer (CT) analyses performed using the combined S K-edge NEXAFS and S–K L_2,3_L_2,3_ RAS core-level spectroscopy techniques, which indicate that the electronic coupling between the S 3p states of WS_2_ and the conduction band electronic states of SLG favor interfacial electron transfer, corroborate with the results of spin-pumping measurements presented in the previous section. This shows that a heterostructure with the combination of WS_2_ and SLG makes the system with increased efficiency both in electronic systems and for use in spintronics and combined devices.

### 3.4 Theoretical Interpretation

**Figure 7:**
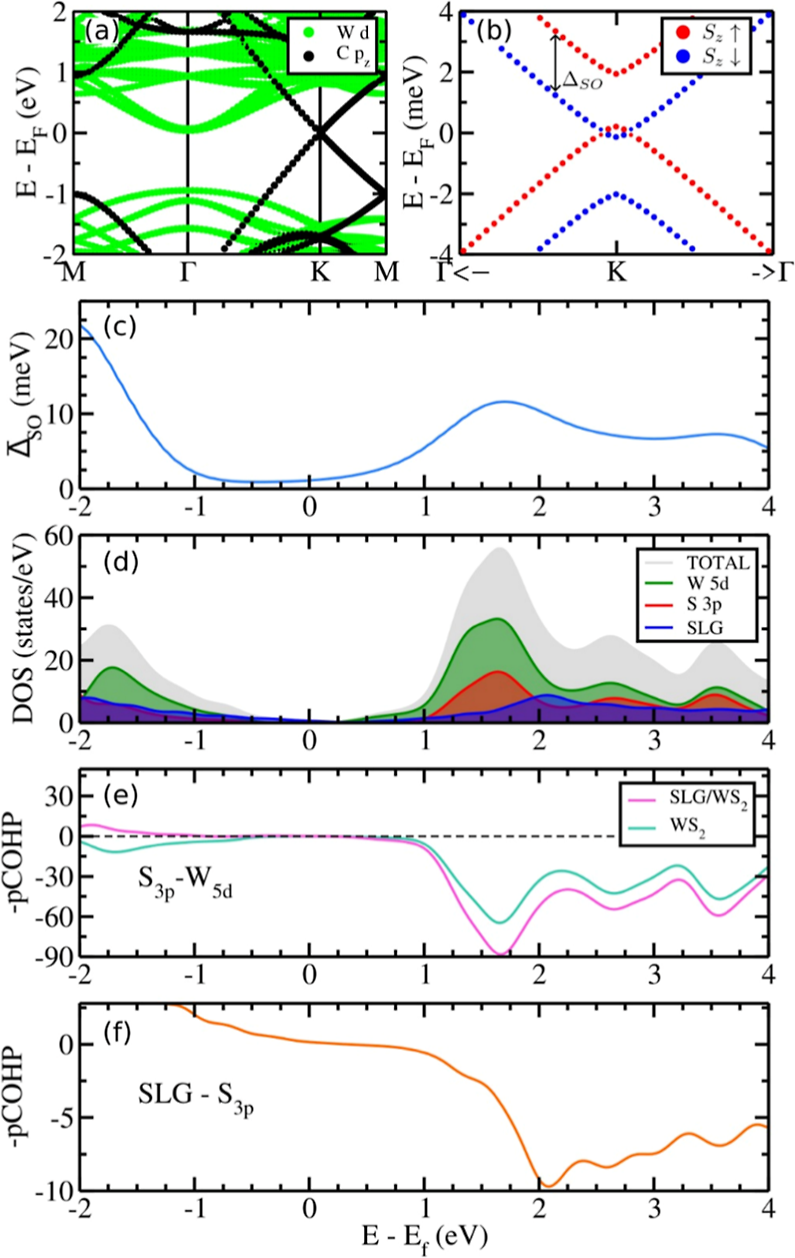
(a) Band structure with projected contributions of SLG (black) and WS_2_ (green) orbitals. (b) Spin-projected band dispersion close to the *K* point. (c) Effective spin orbit splitting  of SLG states. (d) Projected density of states. The crystal orbital Hamilton population processed with LOBSTER software (pCOHP) between (e) 3p orbitals of S and 5d orbitals of W and (f) 3p orbitals of S and SLG states.

In order to understand the mechanisms behind the enhancement of the spin-to-charge conversion on the SGL/WS_2_ heterostructure and the electronic coupling between the conduction band states of graphene and the S 3p states of WS_2_, we performed ab initio calculations. A detailed description of the construction of the SLG/WS_2_ supercell for these calculations is presented in the Supporting Information. Figure 7a shows the electronic structure of the SLG/WS_2_ interface, where the projected orbitals depict the graphene Dirac cone neatly laying within the energy bandgap of WS_2_. Despite the van der Waals (vdW) nature of the interface, WS_2_ induces pronounced spin–orbit (SO) effects on the graphene states. This phenomenon induces spin splitting of the graphene Dirac cone, as depicted in Figure 7b, similar to a Rashba effect. Such characteristic of the Rashba-like spin texture is responsible for the experimentally observed spin-charge conversion.^59^ In the Supporting Information, we discuss the effect of the interface electric field on the increase of the Rashba spin splitting in the SLG/WS_2_ interface (Figure S4). Here, the effective spin splitting is defined by calculating the mean average over the graphene states
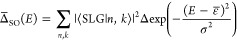
where ⟨SLG|*n*,*k*⟩ is the projected graphene orbitals in the Bloch eigenstates, Δ is the SOC splitting, and ε̅ is the mean energy between the SOC split states. Such a term accounts for not only the SOC splitting but also the graphene contribution and the density of states (DOS). In Figure 7c is presented the effective spin splitting. At the Fermi energy, we find a value of 1.1 meV, while at 1.6 eV above the Fermi energy, the splitting increases 10-fold. This can be understood by the superposition of the WS_2_ states with the graphene pz orbitals. For instance, the projected density of states (Figure 7d) shows that at such an energy window, we have a superposition of W 5d states and S 3p states with the graphene pz orbitals.

Figure 7d displays the total and partial density of states (DOS) for the SLG/WS_2_ heterostructure. In the analysis of Figure 7d, we can observe that W 5d states predominantly constitute the conduction band of the SLG/WS_2_ system. Beyond an energy threshold of 2 eV above the Fermi level, the graphene states gradually become more pronounced. Figure 7e,f exhibits the corresponding crystal orbital Hamilton population (-pCOHP) analysis. A significant electronic coupling between S 3p states and graphene (Cp) states is observed within the energy range 2–4 eV above the Fermi energy of the system, coinciding with a notable increase in the SOC spin splitting parameter. The significant electronic coupling and the increase of the SOC splitting parameter are indicative of the efficient interfacial charge transfer and enhancement of the spin-to-charge conversion process in the SLG/WS_2_ heterostructure.

## 4 Conclusions

In this article, we report spintronic measurements performed via the ferromagnetic resonance technique on structures composed of SLG/Py and (SLG/WS_2_)/Py. From these measurements, we extracted the IREE parameters that quantify the spin-to-charge current conversion by the Rashba–Edelstein mechanism, yielding λ_IREE_^SLG/Py^ = (0.011 ± 0.002) nm and λ_IREE_^(SLG/WS2)/Py^ = (0.078 ± 0.005) nm, respectively. These results highlight a significant enhancement in the conversion of spin current into charge current by a factor of approximately 7 in the presence of WS_2_ in contact with the SLG. We attribute this enhancement primarily to the proximity effect observed in graphene. To better understand this enhancement, we carried out a charge transfer study to investigate the electronic coupling between WS_2_ and graphene. Our results indicate a rapid interfacial electron transfer facilitated by the electronic coupling between the S 3p states of WS_2_ and the conduction band electronic states of SLG within this heterostructure. Additionally, a theoretical binding study was carried out to elucidate the coupling between these electronic states, with the findings corroborating the experimental results. Analysis of the density of states shows that the W 5d states predominantly constitute the conduction band of the SLG/WS_2_ heterostructure. Furthermore, the spin–orbit effect induced by WS_2_ in graphene leads to spin-splitting of the graphene Dirac cone, characteristic of the Rashba effect. In this work, we demonstrate that the spin–orbit coupling and the electronic coupling between the states of graphene and WS_2_ are intrinsically related to each other and that the combination of these phenomena is responsible for the enhancement observed in the spin-to-charge current conversion of the SLG/WS_2_ heterostructure.

## 5 Experimental Section

### 5.1 Raman Spectroscopy

Raman spectra were collected at room temperature using a WITec Alpha 300R confocal Raman imaging microscope using a laser line centered at 532 nm with a power of 0.508 mW.

### 5.2 X-ray Photoelectron Spectroscopy (XPS)

XPS measurements were performed in an ultrahigh vacuum chamber with a base pressure of 10^–8^ mbar using a hemispherical electron energy analyzer (Specs model PHOIBOS 150) with a 45° takeoff direction of electrons and a pass energy of 25 eV. Photon energy calibration was performed using the Au 4f_7/2_ core level at 84.0 eV of a gold foil. The total energy resolution achieved was 0.76 eV. Fittings of XPS spectra (blue and red lines) were performed by using pseudo-Voigt profile functions, with the background correlation performed by using a Shirley function. Surface charge effects, manifesting as shifts in electron energy, were monitored using the C 1s (C–C) photoemission line localized at a binding energy of 285 eV.

### 5.3 Ferromagnetic Resonance (FMR)

Measurements of the ferromagnetic resonance (FMR) were performed in a homemade spectrometer. The samples were attached to the tip of a poly(vinyl chloride) (PVC) rod and inserted into a rectangular microwave cavity through a hole in the center of its back wall. It operated in the transverse electric (*TE*_102_) mode at a frequency of 9.4 GHz with a *Q* factor of 2000. The PVC rod was positioned where the electric field was zero and the rf magnetic field was maximum to avoid galvanomagnetic effects. The microwave cavity was positioned between the poles of an electromagnet, and the sample could be rotated while keeping the static and rf fields in the sample plane and perpendicular to each other. With this configuration, the angular dependence of the FMR absorption spectra can be investigated. Field scan spectra of the derivative of the microwave absorption  were obtained by modulating the dc field with the weak ac field, *h*_ac_(*t*) = *h*_m_ sin(2π*ft*), where *f* = 1.2 kHz and *h*_m_ ≈ 1.0*Oe*. This field served as the reference for lock-in detection of the absorption of the RF field.

### 5.4 Spin Pumping Measurements

The spin pumping measurements were performed in the same setup as the FMR measurements. In the resonance condition, the spin current generated in the ferromagnetic layer by magnetization dynamics was pumped into the heterostructure (adjacent layer). In this layer, the spin current can be converted into a charge current by the inverse spin Hall effect or the inverse spin Rashba effect, and the electrical signal was detected using a nanovoltmeter through electrodes attached with silver paint on the Ti/Au strips. To reduce noise from interferences, the external field modulation used to obtain the FMR spectra was turned off.

### 5.5 X-ray Absorption Near Edge Structure (S K-Edge NEXAFS)

Sulfur K-edge NEXAFS spectra were collected using total electron yield detection mode (TEY), measuring the drain current through the sample. The spectra were measured at the soft X-ray spectroscopy (SXS) beamline at the Brazilian Synchrotron Light Source (LNLS) facility. The NEXAFS spectra were normalized by the photon flux spectrum (estimated from the Au mesh) to correct the fluctuations in the beam intensity. The average NEXAFS spectra were obtained from at least three scans.

### 5.6 Resonant Auger Spectroscopy (S–K L_2,3_L_2,3_ RAS)

The S-KL_2,3_L_2,3_ RAS spectra were collected using specific photon energy around the S–K absorption edge with a hemispherical electron energy analyzer (Specs model PHOIBOS 150) with a pass energy of 25 eV and a 45° takeoff direction of Auger electrons.

## 6 Computational Details

The structural and electronic properties in the present work were performed using first-principles calculations based on density functional theory (DFT),^60,61^ as implemented in the Vienna “ab initio” simulation package, VASP.^62^ The DFT-D2 method of Grimme^63^ was considered for the van der Waals (vdW) interactions in all simulations. More computational details can be found in the Supporting Information.

## Data Availability

The data that support the findings of this study are available from the corresponding authors upon reasonable request.

## Supporting Information Available

Schematic representation of the core-hole clock approach used in charge transfer dynamics analysis; photon energy dependence of the electron kinetic energy of Auger decay channels; computational methods; details on the construction of the SLG/WS_2_ supercell for ab initio calculations; and effect of the interface electric field on the increase of the Rashba spin splitting (PDF)
