# Supplementary material for: Unraveling the Spin-to-Charge Current Conversion Mechanism and Charge Transfer Dynamics at the Interface of Graphene/WS2 Heterostructures at Room Temperature
Source: ACS Appl Mater Interfaces. 2024 Oct 2;16(41):56211–20. doi: 10.1021/acsami.4c08539 (PMC11492317; doi:10.1021/acsami.4c08539)
Supplement: Supplementary file 1 — am4c08539_si_001.pdf [file am4c08539_si_001.pdf]

Supporting Information

Unraveling the Spin-to-Charge Current Conversion Mechanism and Charge Transfer Dynamics at the Interface of Graphene/WS_2 Heterostructures at Room Temperature

Rafael O. Cunha[a*], Yunier Garcia-Basabe[b], Dunieskys G. Larrude[c], Matheus Gamino[d], Erika N. Lima[e,f], Felipe Crasto de Lima[f], Adalberto Fazzio[f], Sergio M. Rezende[g], Antonio Azevedo[g], and Joaquim B. S. Mendes[a]

[a]Departamento de Física, Universidade Federal de Viçosa, 36570-900 Viçosa, Minas Gerais, Brazil
[b]Centro Interdisciplinar de Ciências da Natureza, Universidade Federal da Integração Latino-Americana, 85867-970 Foz do Iguaçu, Paraná, Brazil
[c]Escola de Engenharia, Universidade Presbiteriana Mackenzie, São Paulo 01302-907, Brazil
[d]Departamento de Física, Universidade Federal do Rio Grande do Norte, 59078-900 Natal, Rio Grande do Norte, Brazil
[e]Instituto de Física, Universidade Federal de Mato Grosso, 78060-900 Cuiabá, Mato Grosso, Brazil
[f]Ilum School of Science, Brazilian Center for Research in Energy and Materials (CNPEM), 13083-970 Campinas, São Paulo, Brazil
[g]Departamento de Física, Universidade Federal de Pernambuco, 50670-901 Recife, Pernambuco, Brazil

*Email: rafael.cunha@ufv.br

This Supporting Information is composed by four sections:

- **A- Schematic representation of Core Hole clock approach used to Charge transfer dynamic analysis - Supporting Figure.**
- **B- The photon energy dependence of electron kinetic energy of Auger decay channels.**
- **C- Computational Methods.**
- **D- Details on the construction of the SLG/WS_2 supercell for ab initio calculations.**
- **E- Effect of interface electric field in the increase of the Rashba-spin splitting.**
- **F- References.**

A. Schematic representation of Core Hole clock approach used to Charge transfer dynamic analysis - Supporting Figure.

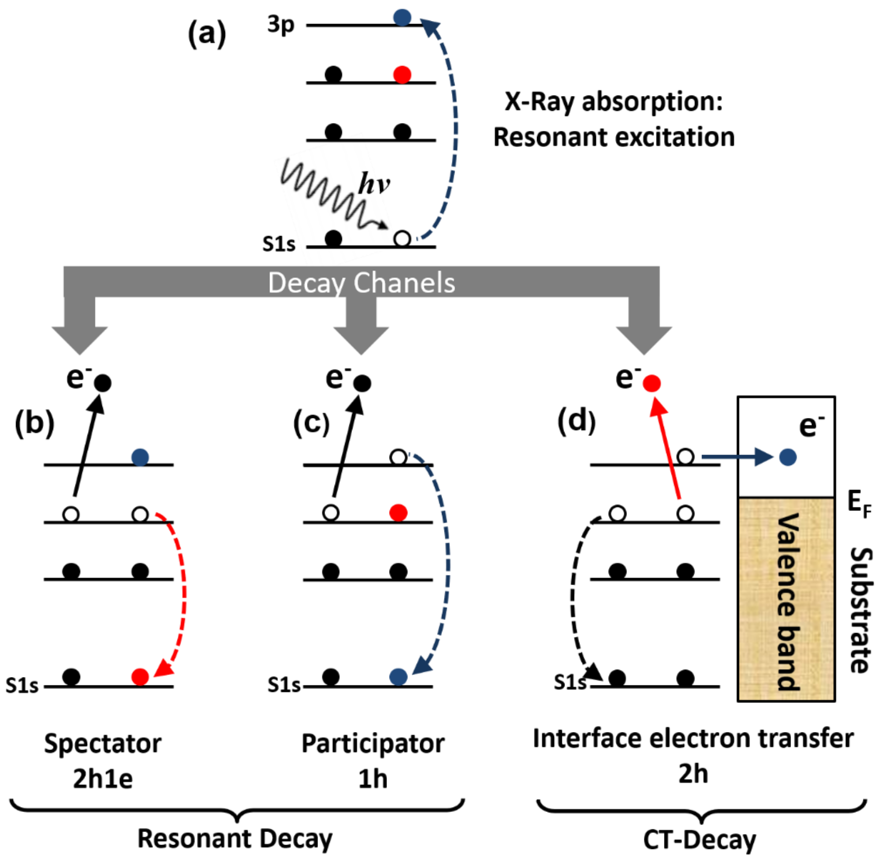

Figure S1. (a) Resonant excitation of a core–electron into an unoccupied electronic state. (b) Spectator Auger decay process resulting in a final state with two holes and one excited electron (2h1e). (c) Participator Auger decay process, leading to a final state with one hole (1h). (d) Fast electron transfer to the substrate (or molecular environment) followed by normal Auger decay, leading to a final state with two holes (2h).

B. The photon energy dependence of electron kinetic energy of Auger decay channels.

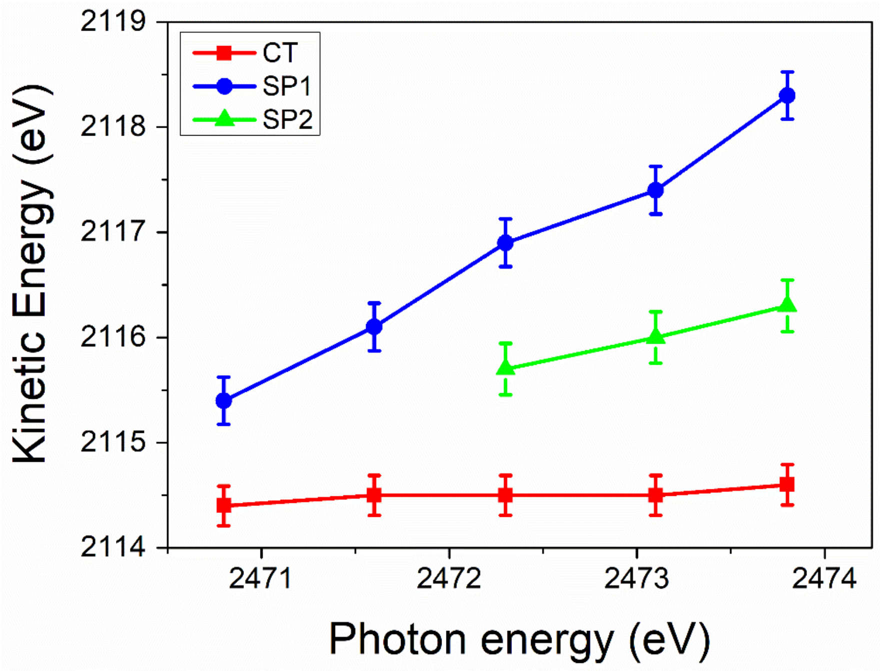

Figure S2. The photon energy dependence of electron kinetic energy for spectator SP1 (blue feature), SP2 (green) and CT (red curve) decay channels.

Table S1. Main parameters obtained from the fitting of sulfur KLL RAS spectra of WS_2/SiO_2 thin film collected at various X-ray photon energies using the sum form (SGL) form Pseudo-Voigt profile functions.

**hv (eV)**	**CT-Auger peak**			**SP1**			**SP2**		
	**KE (eV)**	**FWHM (eV)**	**A (%)**	**KE (eV)**	**FWHM (eV)**	**A (%)**	**KE (eV)**	**FWHM (eV)**	**A (%)**
**2470.8**	**2114.4**	**1.75**	**64**	**2115.8**	**1.55**	**24**	**-**	**-**	**-**
**2473.0**	**2114.4**	**1.79**	**67**	**2117.1**	**1.46**	**12**	**2116.3**	**1.44**	**10.4**

C. Computational Methods.

The structural and electronic properties in the present work were performed using first-principles calculations based on density functional theory (DFT),[1,2] as implemented in the Vienna "ab initio" Simulation Package, VASP.[3] The generalized gradient approximation (GGA) describes the exchange and correlation potentials. The interactions between the valence electrons and the ionic cores are treated within the projector augmented wave (PAW) method.[4,5] The DFT-D2 method of Grimme[6] was considered for the van der Waals (vdW) interactions in all simulations. In the DFT-D2 scheme, the vdW interaction is described by adding a semi-empirical dispersion potential to the conventional DFT energy.

The electronic wave functions are expanded on a plane wave basis with an energy cutoff of 400 eV. Convergence with respect to the energy cutoff was carefully examined using calculations with cutoffs ranging from 400 to 600 eV. The SLG/WS_2 supercells are on the x-y plane, and the Brillouin zone (BZ) integrations are performed using a 10x10x1 Γ-centered Monkhorst-Pack sampling.[7] To avoid interactions between the periodic images of the supercell, the systems are modeled using supercells repeated periodically on the x-y plane with a vacuum region of about 15 Å inserted along the z-direction. For bonding analysis, we utilized the LOBSTER[8,9] tool to evaluate the electronic density of states (DOS) and the crystal orbital Hamilton population (pCOHP).[10,11] Spin-orbit coupling is included in all electronic structure calculations.

D. Details on the construction of the SLG/WS_2 supercell for ab initio calculations.

In this work for ab initio calculations, we use the equilibrium geometry of the SLG/WS_2 heterostructure is depicted in Figure below. This unicell was constructed by stacking a 4x4 supercell of SLG and a 3x3 WS_2 monolayer. The lattice constant of the SLG/WS_2 heterostructure is fixed at that of the 4x4 SLG, while WS_2 monolayer being under a biaxial tensile strain of 3.34 %. It is worth pointing out that such strain does not significantly change the electronic properties of the WS_2 monolayer, such as orbitals hybridization and energy gap.

Moreover, to investigate the structural stability of the SLG/WS_2 heterostructure, three different types of stacking were considered: CS, CW, and center configurations. In the CS and CW configurations, two C atoms of SLG are situated directly above two S and W atoms of the WS_2 monolayer, respectively. For the center configuration, one C atom of SLG is situated in the center of a hexagon of the WS_2 monolayer. Regardless of the adsorption configuration, one can see no appreciable differences in the surface structures of SLG compared to isolated SLG. The WS_2 monolayer induces small corrugation on SLG of 0.015 Å. For all configurations, the distance (d_0) between SLG and the topmost sulfide layer of the WS_2 monolayer is approximately 3.32 Å. Regarding stability, the total energy per cell always favors the CS stacking type, with a difference of 11.0 meV per cell for the CW configuration and 15.1 meV per cell for the center configuration.

The Figure below shows the CS configuration, where (a) top and (b) side views of the optimized geometry of the SLG/WS_2 heterostructure are presented. The brown, grey, and yellow balls represent the C, W, and S atoms. The distance between SLG and WS_2 is denominated d_0.

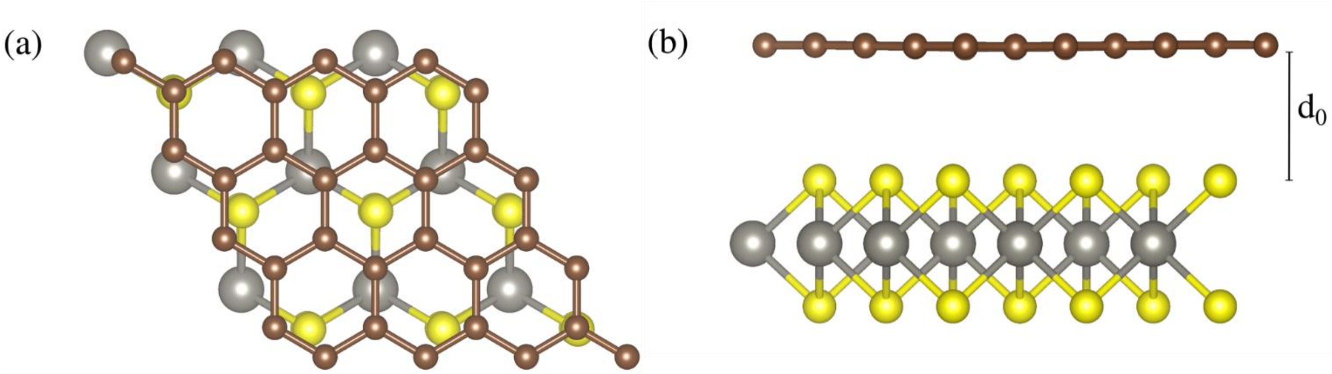

Figure S3. a) Top and (b) side view of the optimized geometry of SLG/WS_2 heterostructure. The brown, grey, and yellow balls represent the C, W, and S atoms. The distance between SLG and WS_2 is denominated d_0.

E. Effect of interface electric field in the increase of the Rashba-spin splitting.

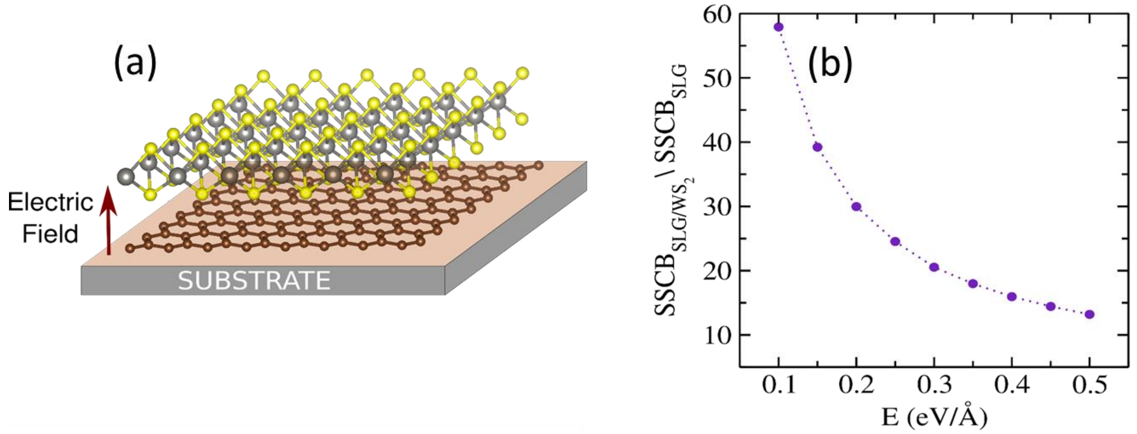

Figure S4. (a) System geometry with the SLG on top of a substrate and the overayed WS_2 system. (b) Effect of an external electric field on the spin-orbit splitting fraction for the system with/without the WS_2.

We investigated the impact of the SiO_2/Si substrate and the effect of other insulating substrates on the spin-orbit splitting character of graphene bands. To assess the substrate effect, we applied a positive low-magnitude external transverse electric field, as depicted in Fig. 7(a), which allowed us to examine the behavior of the heterostructure under intrinsic interface fields. To capture the possible effect of other interfaces, we varied the range of the external transverse electric field in our calculations between 0.1 to 0.5 eV/Å, and the direction of the positive electric field goes from SLG to WS_2.

When an external transverse electric field is applied to the SLG/WS_2 heterostructure, it modifies the electronic band structure, the break of reflection symmetry by the external field introduces a Rashba spin-orbit term. This can lead to changes in the spin-splitting, a property intrinsically linked to spin-charge conversion. Here we can take graphene's spin-splitting of the conduction band (SSCB) as a signature of the spin-charge conversion strength. Figure SI-2(b) illustrates the ratio between the SSCB for the SLG/WS_2 interface and the pristine SLG ($SSCB_{SLG/WS2}/SSCB_{SLG}$) as a function of the external electric field. Pristine SLG without external fields presents zero SSCB; therefore, the SSCB ratio increases for lower fields. The breaking of perpendicular spatial symmetry in the heterostructure increases the spin-charge conversion signature. It's worth noting that even in the higher-field scenario, our research indicates that the SSCB in the SLG/WS_2 heterostructure is 13 times higher than in the isolated SLG. This disparity is attributed to the prominent SOC in the WS_2 and its Rashba-induced effect on SLG.

E. References.

(1) Hohenberg, P.; Kohn, W. Inhomogeneous Electron Gas. *Physical review* **1964**, *136* (3B), B864. https://doi.org/10.1103/PhysRev.136.B864.

(2) Kohn, W.; Sham, L. J. Self-Consistent Equations Including Exchange and Correlation Effects. *Physical review* **1965**, *140* (4A), A1133. https://doi.org/10.1103/PhysRev.140.A1133.

(3) Kresse, G.; Furthmüller, J. Efficient Iterative Schemes for Ab Initio Total-Energy Calculations Using a Plane-Wave Basis Set. *Phys Rev B* **1996**, *54* (16), 11169. https://doi.org/10.1103/PhysRevB.54.11169.

(4) Perdew, J. P.; Burke, K.; Ernzerhof, M. Generalized Gradient Approximation Made Simple. *Phys Rev Lett* **1996**, *77* (18), 3865. https://doi.org/10.1103/PhysRevLett.77.3865.

(5) Kresse, G.; Joubert, D. From Ultrasoft Pseudopotentials to the Projector Augmented-Wave Method. *Phys Rev B* **1999**, *59* (3), 1758. https://doi.org/10.1103/PhysRevB.59.1758.

(6) Grimme, S. Semiempirical GGA-type Density Functional Constructed with a Long-range Dispersion Correction. *J Comput Chem* **2006**, *27* (15), 1787–1799. https://doi.org/10.1002/jcc.20495.

(7) Monkhorst, H. J.; Pack, J. D. Special Points for Brillouin-Zone Integrations. *Phys Rev B* **1976**, *13* (12), 5188. https://doi.org/10.1103/PhysRevB.13.5188.

(8) Nelson, R.; Ertural, C.; George, J.; Deringer, V. L.; Hautier, G.; Dronskowski, R. LOBSTER: Local Orbital Projections, Atomic Charges, and Chemical-Bonding Analysis from Projector-Augmented-Wave-Based Density-Functional Theory. *J Comput Chem* **2020**, *41* (21), 1931–1940. https://doi.org/10.1002/jcc.26353.

(9) Maintz, S.; Deringer, V. L.; Tchougréeff, A. L.; Dronskowski, R. LOBSTER: A Tool to Extract Chemical Bonding from Plane-Wave Based DFT. *J Comput Chem* **2016**, *37* (11), 1030–1035. https://doi.org/10.1002/jcc.24300.

(10) Deringer, V. L.; Tchougréeff, A. L.; Dronskowski, R. Crystal Orbital Hamilton Population (COHP) Analysis as Projected from Plane-Wave Basis Sets. *Journal of Physical Chemistry A* **2011**, *115* (21), 5461–5466. https://doi.org/10.1021/jp202489s.

(11) Maintz, S.; Deringer, V. L.; Tchougréeff, A. L.; Dronskowski, R. Analytic Projection from Plane-Wave and PAW Wavefunctions and Application to Chemical-Bonding Analysis in Solids. *J Comput Chem* **2013**, *34* (29), 2557–2567. https://doi.org/10.1002/jcc.23424.
